# Changing HIV guidelines: how to communicate treatment start

**DOI:** 10.1186/1758-2652-13-S4-P117

**Published:** 2010-11-08

**Authors:** J Fehr, D Nicca, W Langewitz, D Haerry, M Battegay

**Affiliations:** 1 University Hospital Zürich/ University of Zürich, Infectious Diseases and Hospital Hygiene, 8091 Zürich, Switzerland; 2 Cantonal Hospital St. Gallen, Infectious Diseases and Hospital Hygiene, 9007 St. Gallen, Switzerland; 3 University Hospital Basel/ University of Basel, Psychosomatic Medicine/ Internal Medicine, 4031 Basel, Switzerland; 4 University Hospital Basel/ University of Basel, Infectious Diseases and Hospital Hygiene, 4031 Basel, Switzerland

## Purpose of study

Recent treatment guidelines support the start of antiretroviral treatment (ART) in HIV-infected persons already at a CD4-cell count threshold 350 cells/µl. This includes to a large proportion asymptomatic individuals who do not necessarily see a good reason to start ART. In such cases, counselling can become a challenge. There is a lack of structured tools to optimally assess patients' readiness and to support them in this process. The purpose of this project was twofold: First to develop an algorithm for health care providers (HCP) to guide patients in the situation of treatment start. Second to develop a workshop during which HCP are instructed how to implement the algorithm and how to improve communication skills.

## Methods

Based on an action research approach, consecutively literature review, own quantitative and qualitative studies and expert panel discussions were performed. We developed an algorithm and piloted an educational program for HCP. For this program critical incident reporting by experienced HIV providers was used and usability was evaluated in two workshops with HCP (self-reporting and group feedback).

## Results

The readiness counselling algorithm has been integrated into updated European guidelines (http://www.europeanaidsclinicalsociety.org/guidelines.asp). It takes into account that patients are at different stages of readiness to start ART and that there are barriers (e.g. depression) before starting ART which have to be identified. An assessment of patients' actual stage of readiness and stage-based decision making support is recommended. The pilot workshop uses techniques of patient-oriented communication (waiting, echoing, mirroring, summarising) and a video-based interaction module, in which HCP present individual patients in whom the initiation of ART proved to be difficult. Re-playing these short case vignettes gives all participants a chance to apply newly acquired communication techniques. Participants rated these workshops very positive, emphasizing the high degree of practicality, closeness to their daily work, and usefulness of communication tools.

## Conclusion

We developed an algorithm to assess and improve patients' readiness to start ART and a corresponding workshop on the use of the algorithm. Pilot workshops show that the algorithm is easy to implement into daily practice, shows excellent acceptance and provides a basis for the successful initiation of ART and long-term adherence to treatment.

